# Interactive effects of temperature and bisphenol A on phytoplankton growth and community structure

**DOI:** 10.1093/conphys/coad021

**Published:** 2023-05-03

**Authors:** Meredith E Theus, Julia Michaels, Samuel B Fey

**Affiliations:** Department of Biology, Reed College, 3203 SE Woodstock Blvd, Portland, OR 97202, USA; Department of Biology, Reed College, 3203 SE Woodstock Blvd, Portland, OR 97202, USA

**Keywords:** Phytoplankton, Environmental contamination, Climate warming

## Abstract

Environmental contamination of bisphenol A (BPA) is a widespread and multifaceted issue with vast ecological, social and economic consequences. Thus, understanding how local environmental conditions, such as temperature, interact with BPA to affect populations and community dynamics remain important areas of research. Here, we conduct laboratory experiments aimed at understanding how environmental gradients of both temperature and BPA concentration influence freshwater phytoplankton population growth and community structure. We exposed phytoplankton assemblages comprised of three common species of green algae (*Chlorella vulgaris, Ankistrodesmus braunii* and *Scenedesmus quadricauda*) as well as isolates of each individual species to three BPA concentrations (0, 2, 13 mg/L BPA) and three temperatures (18, 23, 27°C) monitoring population growth and community structure (via biovolume). We observed antagonistic interactions between BPA and warmer temperatures, such that when warmer temperatures decreased growth (observed with *A. braunii*), high concentrations of BPA elevated growth at these warm temperatures; however, when warmer temperatures increased growth (*C. vulgaris*, *S. quadricauda*), high BPA concentrations diminished these gains. Although BPA exposure inhibited the growth of most *C. vulgaris* populations, growth was not reduced in *A. braunii* or *S. quadricauda* populations exposed to 2 mg/L BPA. Phytoplankton assemblage evenness (Pielou evenness index) decreased as BPA concentration increased and was consistently lowest under 27°C. Community composition was similar in assemblages cultured under 0 and 2 mg/L BPA under 18 and 23°C but was most similar between assemblages cultured under 2 and 13 mg/L BPA under 27°C. These results indicate that local environmental temperatures can mediate the consequences of BPA for freshwater phytoplankton growth rates and community structure and that BPA can diminish potential gains of increased growth rate for warm-adapted phytoplankton species at high environmental temperatures.

## Teaser

We investigate how environmental temperature and bisphenol A (BPA, a widespread environmental contaminant) jointly alter algal populations and communities. We report that higher environmental temperature can exacerbate or ameliorate the negative impacts of BPA on growth rates and may increase the sensitivity of algal community composition to BPA.

## 1. Introduction

Environmental contamination of freshwater ecosystems is a pervasive, multifaceted issue that affects ecological, social and economic systems (Schwarzenbach *et al.,* 2010; Amoatey and Baawain, 2019). The introduction of novel or rare compounds to the environment has resulted in increased concentrations of biologically unavailable forms of carbon and nitrogen that accumulate in ecosystems (Vitousek *et al.,* 1997) with, in many instances, undesired consequences for organisms, populations and communities. Certain pollutants may reduce the fitness of individuals and subsequently the growth of populations by interacting with and potentially altering metabolic pathways, hormone signaling and enzyme activity (Canesi and Fabbri, 2015; Xiang *et al.,* 2018; Wu and Seebacher, 2020). Importantly, the metabolic and demographic rates of the individuals and populations interacting with contaminants are also influenced by local environmental conditions (Staples *et al.,* 1998; Savage *et al.,* 2004; Borrirukwisitsak *et al.,* 2012; Dell *et al.,* 2013; Wu and Seebacher, 2020). In certain instances, abiotic factors may influence the toxicity threshold as well as the effects a given contaminant has on organisms (Little and Seebacher, 2015). In the following manuscript, we investigate the joint effects of a ubiquitous contaminant ((bisphenol A (BPA)) and water temperature on phytoplankton growth rates and community structure to understand the impact of continued environmental contamination and continued climate warming on aquatic ecosystems.

BPA is a compound commonly used in the manufacturing of household products, medical equipment and food storage equipment, and it is widespread in aquatic ecosystems. Surface water concentrations of BPA are highly variable (Wu and Seebacher, 2020), and there are higher average BPA levels in freshwater systems (reported BPA concentrations range from 0 to 63.64 μg/L (Inam *et al.,* 2019; Wu and Seebacher, 2020)) than in estuaries or marine systems (reported BPA concentrations range from 0 to 1.92 μg/L (Bayen *et al.,* 2016; Wu and Seebacher, 2020)). BPA is taken up by aquatic organisms and transferred across trophic levels, allowing for moderate bioaccumulation via trophic transfer as observed in some fish species (Corrales *et al.,* 2015; Wu and Seebacher, 2020). The effects of BPA on individual organisms have been widely studied; however, most of the literature has focused on the effects of acute, single-generation exposure among vertebrates (Flint *et al.,* 2012; Wu and Seebacher, 2020). In certain freshwater species, BPA negatively affects development, behavior and survival (Wu and Seebacher, 2020). It mimics the female estrogen 17β-estradiol and may act more generally as an androgen antagonist as well as a thyroid hormone antagonist, affecting processes beyond reproduction and development, such as growth and metabolism (Crain *et al.,* 2007; Canesi and Fabbri, 2015). By contrast, there are fewer studies and a lack of consensus on the effects, mechanisms of action and metabolism of BPA in invertebrates (Wu and Seebacher, 2020). Yet, the divergent metabolic and hormone signaling pathways and the distinct microhabitats occupied by invertebrates may put certain invertebrate species at a high risk of experiencing the deleterious effects of BPA (i.e. decreased reproduction and growth) (Oehlmann *et al.,* 2009; Flint *et al.,* 2012). BPA is a hydrophobic, organic pollutant that tends to accumulate in cellular lipids, inducing lipid peroxidation and oxidative stress, and it may inhibit photosynthesis and the biosynthesis of chlorophyll (Ji *et al.,* 2014; Li *et al.,* 2018). Unicellular, aquatic species, such as phytoplankton, have few physical barriers between the cellular interior and the environment, and many phytoplankton species readily uptake BPA molecules with consequences to growth and morphology (Gattullo *et al.,* 2012; Ji *et al.,* 2014; Xiang *et al.,* 2018). For example, in the green algae *Scenedesmus quadricauda*, exposure to 1 mg/L BPA can reduce growth, cytoplasm volume and colony size (Xiang *et al.,* 2018).

Environmental temperature (in addition to light, pH and nutrient availability) can also have substantial effects on phytoplankton populations by altering cell division and death rates, such that moderate increases in temperature can accelerate or drastically reduce growth rates (Brown *et al.,* 2004; Ras *et al.,* 2013; Kremer *et al.,* 2017). Exposure to temperatures above the thermal optimum (temperature at which the maximum growth rate is achieved) can cause heat stress leading to the inactivation or denaturation of enzymes that are necessary for photosynthesis and other metabolic reactions, thus slowing rates of population growth and altering productivity, standing biomass and/or phenological responses across seasons (Thomas *et al.,* 2012; Ras *et al.,* 2013). In addition, environmental conditions can influence the rates of abiotic reactions that transform or degrade BPA. Reaction rate, favorability and spontaneity are dependent on temperature in both abiotic and biotic systems, and most biological rates, including metabolic rates, are modified by environmental temperature (Brown *et al.,* 2004). Temperature influences the rates of BPA uptake, metabolism and excretion with the rates of these processes typically increasing with increasing temperature (Honkanen and Kukkonen, 2006; Wu and Seebacher, 2020; Wu and Seebacher, 2021). Thus, temperature may have the capacity to influence the way in which BPA affects individuals and the way in which individuals take up environmental BPA. However, much of the research investigating the effects of BPA on phytoplankton has focused on the remediative capacity of populations while neglecting the effects of BPA on phytoplankton communities.

The extent to which the ecological consequences of BPA on phytoplankton can be modified by local environmental factors is currently unresolved yet seems important given the ecological significance of phytoplankton (Field *et al.,* 1998). Existing data suggest the potential for environmental conditions to modify the consequences of BPA for phytoplankton. For example, whereas *S. quadricauda* growth is inhibited by 1 mg/L BPA under moderate light intensities in fluctuating light regimes (Xiang *et al.,* 2018), growth of *S. quadricauda* is not inhibited by 10 mg/L BPA under a high, continuous irradiance level (Nakajima *et al.,* 2007). Evidence also exists that environmental temperature can mediate the effects of BPA on other organisms. For example, BPA may synergistically interact with temperature to reduce thermal tolerance in *Danio rerio*, zebrafish (Little and Seebacher, 2015). However, the relationship between BPA and temperature may not be as clear in all cases and can depend on both past and current thermal environments (e.g. Wu and Seebacher, 2021). In addition, temperature synergistically interacts with other environmental contaminants besides BPA, including heavy metals (Oukarroum *et al.,* 2012) and certain herbicides (Delorenzo *et al.,* 2013), to negatively affect phytoplankton.

This present study aims to better understand how natural phytoplankton assemblages might respond to continued environmental contamination and climate warming. We address the question of how phytoplankton population growth rates, colony size and community structure are influenced by BPA contamination across a gradient of thermal environments. We hypothesize that BPA and temperature synergistically interact to influence phytoplankton population growth rates, colony size and community evenness such that warmer temperatures will exacerbate any observed detrimental consequences of BPA due to increased rates of BPA uptake cooccurring with heat-induced stress.

## 2. Methods

### 2.1. Study species

The following species of freshwater green algae were used in the experiments subsequently described: *Chlorella vulgaris* (family *Oocystaceae*; University of Texas, UTEX, Culture Collection of Algae, Austin, TX, UTEX #26)*, Ankistrodesmus braunii* (family *Selenastraceae*; UTEX #245) and *S. quadricauda* (family *Scenedesmaceae*; UTEX #B 76). Species were chosen based on their demonstrated ability to remove BPA from the environment (Nakajima *et al.,* 2007; Gattullo *et al.,* 2012; Ji *et al.,* 2014), their presence in freshwater environments (U.S. EPA, 2021) and their unique morphologies to allow for distinction between species. We anticipated that *A. braunii* would have a reduced tolerance to high environmental temperatures based on previous laboratory assays (Talbot *et al.,* 1991; Kremer *et al.,* 2017). Before experimentation, phytoplankton populations of each species were maintained under continuous light (~60 μmol·m^−2^·s^−1^) and a constant temperature (20°C) in a COMBO nutrient solution (Kilham *et al.,* 1998). *Ankistrodesmus braunii* and *S. quadricauda* cultures were maintained for 3 months before experimentation, and *C. vulgaris* cultures were maintained for 12 months before experimentation.

Cultures were started and maintained using sterile techniques. Algal cultures were unialgal when purchased from The University of Texas Algal Culture Collection, and the COMBO media was initially sterilized by autoclaving at 121°C for 1 hour. Moreover, before experimentation, cultures of each species were imaged using a FlowCam 5000 (Yokogawa Fluid Imaging Technologies, Inc., Scarborough, ME) to develop species-specific filters to be used in the experiment (discussed in *Laboratory Methods: Phytoplankton Community Assay*), and there was no observed contamination. Phytoplankton were cultured in a COMBO nutrient solution (Kilham *et al.,* 1998) before and during experimental assays and maintained under continuous light at 94.8 ± 17.4 μmol·m^−2^·s^−1^ (mean ± 1 SD) on a thermal gradient block (TGB) (Fey *et al.,* 2021) for the entirety of the experiment.

### 2.2. Laboratory methods: Phytoplankton population assay

We performed an experiment from January to March 2021 to measure the effects of temperature and BPA on phytoplankton population growth referred to as the ‘population assay’ (containing a single species).

Phytoplankton populations were acclimated to their experimental temperature (18, 23, 27°C) >1 week and maintained in an exponential growth phase to ensure that any observed changes in growth were due to the treatments and were not confounded by density-dependent effects. This duration of acclimation time was previously observed to be adequate for the growth rates of phytoplankton to adjust to novel thermal environments (Fey *et al.,* 2021). Experimental temperatures were chosen to include two temperatures (18 and 23°C) that phytoplankton commonly experience in temperate ecosystems (Zargar *et al.,* 2006; Serra-Maia *et al.,* 2016; U.S. EPA, 2021; Layden *et al.* 2021) and a potentially stressful but non-lethal temperature of 27°C that may become more likely to occur as lake heat waves intensify due to climate warming (Woolway *et al.,* 2021). Collectively these three temperatures (18, 23, 27°C) provide a gradient of thermal environments that phytoplankton can experience or may experience in the future for extended periods. Phytoplankton (*C. vulgaris, A. braunii, S. quadricauda*) were subsequently exposed to the constant temperature to which they were acclimated and one of three BPA concentrations (0, 2, or 13 mg/L BPA). BPA (CAS: 80–05-7; Sigma-Aldrich, St. Louis, MO) concentrations were chosen to be comparable to those used in other experiments (Nakajima *et al.,* 2007; Gattullo *et al.,* 2012; Ji *et al.,* 2014; Xiang *et al.,* 2018). Although reported average BPA concentrations in natural bodies of water reach up to 63.64 μg/L (Inam *et al.,* 2019; Wu and Seebacher, 2020), BPA concentrations in landfill leachates have been reported to be >17 mg/L (Yamamoto *et al.,* 2001), which even exceeds those tested in this experiment. Although pulse events may result in momentarily high concentrations of BPA being introduced to freshwater environments, BPA concentrations of 13 mg/L—although similar to commonly used experimental concentrations in phytoplankton—exceed the scope of typical BPA concentrations commonly recorded in nature.

Phytoplankton cultures were exposed to a single temperature and to either 0, 2 or 13 mg/L BPA (CAS: 80–05-7; Sigma-Aldrich) in a total volume of 3.5 mL COMBO nutrient solution on the TGB for 6 days (n = 3 replicates (Nakajima *et al.,* 2007)). A 6-day assay period was chosen because 6 days was a sufficient period to observe the effects of BPA on the phytoplankton (Nakajima *et al.,* 2007; Gattullo *et al.,* 2012; Ji *et al.,* 2014) without observing large declines in the phytoplankton populations due to density-dependent effects. There were no observed decreases in volume in any of the cultures over the 6-day assay period. Thermally acclimated populations of each species were transferred to glass test tubes at equal starting fluorescence values for each experimental population. Fluorescence values, in relative fluorescence units (RFU), of each culture was measured using a Trilogy Laboratory Fluorometer (Turner Designs, Inc, San Jose, CA) as a proxy for cell density and a chlorophyll-a *in-vivo* module. Fluorescence was measured before incubating the phytoplankton populations (day 0) then once every 24 hours until day 4 for *A. braunii* and *S. quadricauda* populations and day 3 for *C. vulgaris* populations, at which point the cultures exhibited signs of density-dependent growth. Population growth rates were determined by calculating the slope of log fluorescence over time using the growthTools package in R version 4.1.0 (Kremer, 2020; R Core Team, 2020).

### 2.3. Laboratory methods: Phytoplankton community assay

We performed an experiment from January to March 2021 to measure the effects of temperature and BPA on phytoplankton community structure, hereafter referred to as the ‘community assay’ (containing multispecies assemblages).

Phytoplankton cultures were exposed to a single temperature and to either 0, 2 or 13 mg/L BPA (CAS: 80–05-7; Sigma-Aldrich) in a total volume of 3.5 mL COMBO nutrient solution on the TGB for 6 days (n = 3 replicates (Nakajima *et al.,* 2007)). There were no observed decreases in volume in any of the cultures over the 6-day assay period. Multispecies assemblages were seeded with roughly equal biovolumes for each thermally acclimated (see *Laboratory Methods: Phytoplankton Population Assay* for the description of the acclimation process) species (comprised of 34.8% *A. braunii*, 29.7% *C. vulgaris* and 35.6% *S. quadricauda* for all samples) for a total biovolume roughly equal to that of the starting biovolumes of each single-species population. After a 6-day growth period, multispecies assemblages were homogenized by vortexing samples at a low speed, and 0.5 mL of each of the assemblages was assayed using a FlowCam 5000 (Yokogawa Fluid Imaging Technologies, Inc.) at ×100 magnification with a flow rate of 0.3 mL/min using a 100 μm × 2 mm flow cell and an autoimaging rate of 20 frames per second. The flow cell was cleared with 3 mL of DI water between each sample, and the VisualSpreadsheet program (Yokogawa Fluid Imaging Technologies) was used to create classification criteria for each species and assign species identities to each imaged particle. Using filters based on image characteristics and species morphology (i.e. particle area, length and volume), the captured images for all cultures were then classified as one of the following: *A. braunii* single cell, *A. braunii* multiple cells, *S. quadricauda*, *C. vulgaris* 1–4 cells or *C. vulgaris* 4+ cells. Each classified image was also manually checked to ensure that no cells were incorrectly characterized, and if a given image was misclassified, it was moved to the proper classification. The images that were not originally classified or did not match any species filter were filtered (edge gradient ≥50) and sorted by edge gradient (a proxy for the clarity of the image; the distinction between the particle and the background). The images were then manually analysed; thus, images with low resolution or irregular shapes were removed from the run because these particles were not distinguishable as a particular species. From each sample run, we determined the average colony biovolume (individual and multiple cells of the same species) and percent biomass of each species. Biovolume of each cell was determined using species-specific volume equations based on the geometric form associated with each species (Sun and Liu, 2003; biovolume calculations shown in Supplementary Table S1).

All treatments were performed in triplicate except the multispecies assemblages cultured under 23°C and 13 mg/L BPA for the community assay, where a single replicate was lost due to file corruption. This lost sample represented ~1% of the 108 samples from this experiment.

### 2.4. Data analysis

For the population assay, a three-way analysis of variance (ANOVA) test was initially used to determine if temperature, BPA concentration, species identity or the interaction of these factors affected phytoplankton growth rate or colony biovolume. Two-way ANOVA was then used to determine if temperature, BPA concentration or the interaction of temperature and BPA concentration affected the growth rate or colony biovolume of each individual species. For the community assay, Pielou evenness index was determined for each multispecies assemblage based on final biovolumes of each species. Because all phytoplankton species were present in all multispecies assemblages at the conclusion of the assay, evenness was used to describe the assemblages rather than a diversity index that would have additionally incorporated species richness. Two-way ANOVA on log-transformed (see assumptions of statistical test that follows) Pielou evenness index values was used to determine if temperature, starting BPA concentration or the interaction of temperature and starting BPA concentration affected Pielou evenness. We additionally used two-way ANOVA to determine whether BPA concentration and temperature jointly altered the community biovolume at the end of the day 6 growing period. To visualize the differences between community assemblages in response to temperature and BPA, we related phytoplankton community structure to temperature and BPA concentration using Canonical Correspondence Analysis (CCA) and a permutation test with N = 999 permutations. Before conducting the CCA, species abundance was logarithmically transformed due to the large abundance of *C. vulgaris* cells.

Although all population data (growth rates of each species) met the ANOVA assumptions of normality and homogeneity of variance of the data (using Kolmogorov–Smirnov test and a Levene test, respectively), we log-transformed the assemblage-level variable of Pielou evenness to meet these assumptions. In all instances, results were determined to be statistically significant based on α = 0.05, and all statistical analyses were completed using R version 4.1.0 (R Core Team, 2020).

## 3. Results

### 3.1. Phytoplankton population growth

**Figure 1:**
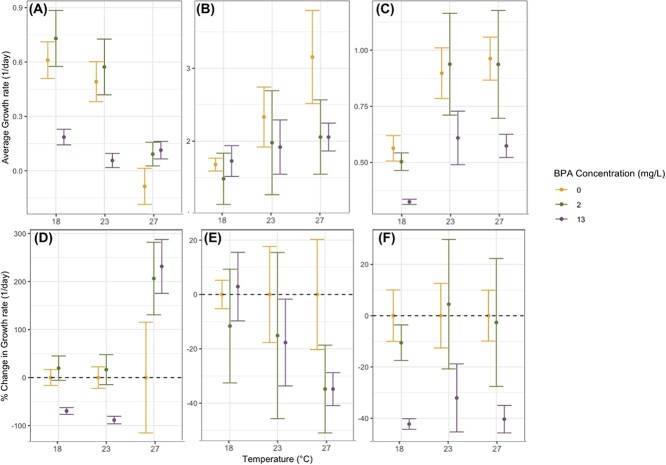
Mean growth rates (1/day) and the percent change (% change) in mean growth rate relative to growth at 0 mg/L BPA for (**A**; **D**) *A. braunii*, (**B**; **E**) *C. vulgaris* and (**C**; **F**) *S. quadricauda* cultured under various combinations of temperature (°C) and BPA concentration (mg/L) with n = 3 replicates. Percent change values in growth rates (**D-F**) were scaled to the average growth rate at 0 mg/L BPA under each temperature. The dashed lines in **D-F** represent no change in growth rate relative to this average. Error bars represent 95% confidence intervals. Note that *y*-axis scales differ across panels.

Phytoplankton population growth rates were influenced by temperature, starting BPA concentration, species identity and their interactions with both shared and divergent patterns of response across species (Figure 1; Supplementary Table S2). When warmer temperatures increased growth rates (as was the case for *S. quadricauda* and *C. vulgaris*) we observed BPA having a damping effect on phytoplankton growth rates (Figure 1, Table 1); yet, when warmer temperatures decreased growth rates (as was the case for *A. braunii*), we observed BPA ameliorating these decreases. Across the three species, the highest growth rates for *A. braunii* (μ = 0.73 ± 0.15 1/day) (average ± 95% confidence interval) and *S. quadricauda* (μ = 0.96 ± 0.10 1/day) in any treatment were less than even the slowest growing *C. vulgaris* cultures (all μ > 1/day).

**Table 1:** Two-way ANOVA results for each species in the population assay

**Species**	**Predictor**	***df***	**Sum square**	**Mean square**	***F***	***P***
*C. vulgaris*	BPA concentration	2	1.628	0.8141	5.524	0.013
	Temperature	2	2.844	1.4219	9.649	0.001
	Temperature × BPA	4	1.184	0.2961	2.009	0.136
	Residuals	18	2.652	0.1474		
*S. quadricauda*	BPA concentration	2	0.3499	0.1749	9.717	0.001
	Temperature	2	0.9245	0.4622	25.672	<0.001
	Temperature × BPA	4	0.0578	0.0145	0.803	0.539
	Residuals	18	0.3241	0.0180		
*A. braunii*	BPA concentration	2	1.1387	0.5694	77.95	<0.001
	Temperature	2	1.0720	0.5360	73.38	<0.001
	Temperature × BPA	4	0.9675	0.2419	33.11	<0.001
	Residuals	18	0.1315	0.0073		

In *A. braunii* populations, the experimental temperature that maximized growth was 18°C. In contrast, *C. vulgaris* and *S. quadricauda* experienced their mean maximum growth rates at 27°C (Figure 1A-C) such that growth rate increased with temperature. *Ankistrodesmus braunii* mean growth rates were consistently higher in 2 mg/L BPA relative to 0 mg/L BPA (Figure 1A); however, the effects of 13 mg/L BPA varied across temperatures. *Ankistrodesmus braunii* growth in populations cultured under 27°C and 13 mg/L BPA was on average 2-fold (231.49 ± 56.05%) greater than populations in 27°C and 0 mg/L BPA, whereas growth was reduced in populations cultured under 18°C and 23°C and 13 mg/L BPA (Figure 1D).

In the absence of BPA, *C. vulgaris* growth increased as water temperature increased such that populations grew slowest under 18°C (Figure 1B, Table 1). *Chlorella vulgaris* growth was significantly affected by BPA concentration yielding the largest reductions in growth under 27°C (34.83 ± 6.06%), whereas no significant impact of BPA was observed under 18°C (Figure 1E; Table 1). Although not statistically significant, populations cultured under 18°C in 13 mg/L BPA grew slightly (2.90 ± 12.62%) faster than in 0 mg/L BPA (Figure 1E). The highest overall *C. vulgaris* growth rate occurred in 0 mg/L BPA.

Temperature and BPA concentration both had significant effects on *S. quadricauda* growth, and in the absence of BPA, *S. quadricauda* cultures grew faster at 23°C and 27°C compared with 18°C (Figure 1C; Table 1). The reductions in growth from 13 mg/L BPA were equally substantial across temperatures, and the single greatest percent reduction in *S. quadricauda* growth rate relative to populations cultured in 0 mg/L BPA occurred in populations exposed to 13 mg/L BPA under 27°C (Figure 1F). Although not statistically significant, populations exposed to 2 mg/L BPA under 23°C were the only treatment to exhibit a subtle increase in growth rate (4.47 ± 25.24%) relative to 0 mg/L BPA treatment. The complete population statistical analyses for each species are reported in Table 1.

### 3.2. Phytoplankton community structure

**Figure 2:**
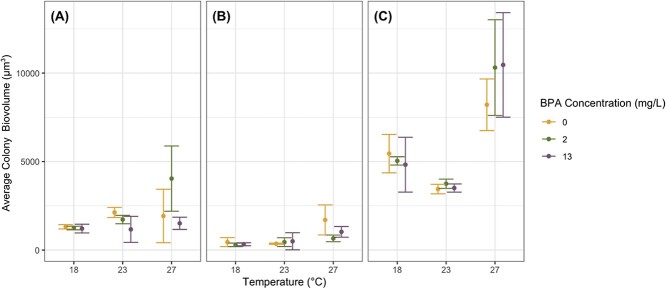
Colony biovolumes (μm^3^) for (**A**) *A. braunii*, (**B**) *C. vulgaris* and (**C**) *S. quadricauda* in the multispecies assemblages cultured under various combinations of temperature (°C) and BPA concentration (mg/L) with n = 3 replicates. Colors are consistent with Figure 1. Error bars represent 95% confidence intervals.

Phytoplankton colony biovolume in the multispecies assemblages was influenced by temperature, species identity and their interaction with colony size generally increasing with temperature (Figure 2; Supplementary Table S3). *Ankistrodesmus braunii* colony biovolume was influenced by temperature, BPA concentration and their interaction, although the effects of BPA concentration were most apparent under 23°C and 27°C (Figure 2A; Table 2). For example, the largest *A. braunii* particles were in assemblages cultured in 0 mg/L BPA under 23°C (2125.89 ± 285.18 μm^3^) and in assemblages cultured in 2 mg/L BPA under 27°C (overall largest *A. braunii* particles; 4037.82 ± 1841.57 μm^3^). *Chlorella vulgaris* and *S. quadricauda* biovolumes in the multispecies assemblages were influenced only by temperature (Figure 2; Table 2). Average *C. vulgaris* colony biovolume was similar across BPA concentrations in 18°C and 23°C and was the largest in assemblages cultured in 0 mg/L BPA and 27°C (1703.26 ± 849.09 μm^3^). *Scenedesmus quadricauda* colony biovolume was seemingly most affected by temperature of the three species with the largest colony biovolumes occurring in assemblages cultured in 27°C (overall largest *S. quadricauda* particles in 13 mg/L BPA; 10 459.92 ± 2952.86 μm^3^) and the smallest colony biovolumes occurring in 23°C (overall smallest *S. quadricauda* particles in 0 mg/L BPA; 3445.50 ± 269.28 μm^3^). However, *S. quadricauda* colony biovolume was similar across BPA concentrations except in 27°C and 0 mg/L BPA.

**Table 2:** Two-way ANOVA results for the average colony biovolume for each species in the community assay

**Species**	**Predictor**	***df***	**Sum square**	**Mean square**	***F***	***P***
*C. vulgaris*	BPA concentration	2	615 943	307 971	3.024	0.0752
	Temperature	2	3 265 624	1 632 812	16.033	0.0001
	Temperature × BPA	4	1 119 288	279 822	2.748	0.0626
	Residuals	17	1 731 310	101 842		
*S. quadricauda*	BPA concentration	2	3 813 133	1 906 567	0.945	0.408
	Temperature	2	17 223 783	86 118 917	42.706	<0.001
	Temperature × BPA	4	7 910 020	1 977 505	0.981	0.444
	Residuals	17	34 281 557	2 016 562		
*A. braunii*	BPA concentration	2	4 443 340	2 221 670	3.847	0.042
	Temperature	2	7 074 993	3 537 497	6.125	0.009
	Temperature × BPA	4	7 568 360	1 892 090	3.276	0.037
	Residuals	17	9 818 574	577 563		

**Figure 3:**
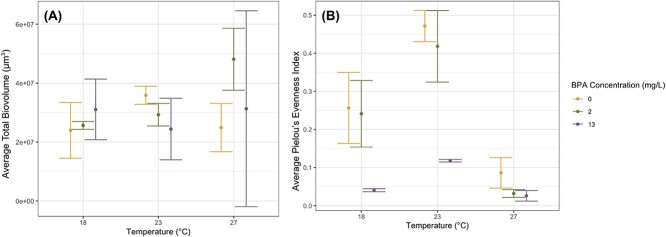
**(A)** Average total biovolume (μm^3^) per 1 mL of sample and **(B)** average Pielou evenness index for multispecies assemblages cultured under unique combinations of temperature (°C) and starting BPA concentration (mg/L). Error bars represent 95% confidence intervals.

In contrast to population growth rate, total assemblage biovolume was not influenced by temperature, BPA concentration or their interaction (Figure 3A; two-way ANOVA BPA, F_2,17_ = 0.646, *P* = 0.537; two-way ANOVA Temperature, F_2,17_ = 0.979, *P* = 0.396; two-way ANOVA BPA × Temperature, F_4,17_ = 1.608, *P* = 0.218). Each phytoplankton species was present in all assemblages at the conclusion of the experiment (species richness = 3), and across all experimental treatments, *C. vulgaris* populations were highly dominant (comprising >80% of the multispecies assemblage biomass).

Evenness (Pielou evenness index) of the multispecies assemblages was affected by temperature, BPA concentration and their interaction (Figure 3B; two-way ANOVA BPA, F_2,17_ = 52.7, *P* < 0.001; two-way ANOVA Temperature, F_2,17_ = 79.3, *P* < 0.001; two-way ANOVA BPA × Temperature, F_4,17_ = 4.06, *P* = 0.017; Supplementary Table S4). Across all BPA concentrations, Pielou evenness index values were highest in assemblages cultured under 23°C and lowest in assemblages cultured under 27°C. Similarly, Pielou evenness index values were consistently highest in assemblages cultured in 0 mg/L BPA and consistently lowest in assemblages cultured under 13 mg/L BPA, with the overall lowest values occurring under both 27°C and 13 mg/L BPA (evenness = 0.03 ± 0.01). Pielou evenness index values were similar between assemblages cultured in 0 mg/L BPA and in 2 mg/L BPA under 18°C and 23°C; however, under 27°C, evenness and community structure were more similar between multispecies assemblages cultured under 2 mg/L and 13 mg/L BPA than assemblages cultured under 2 mg/L BPA and in 0 mg/L BPA.

**Figure 4:**
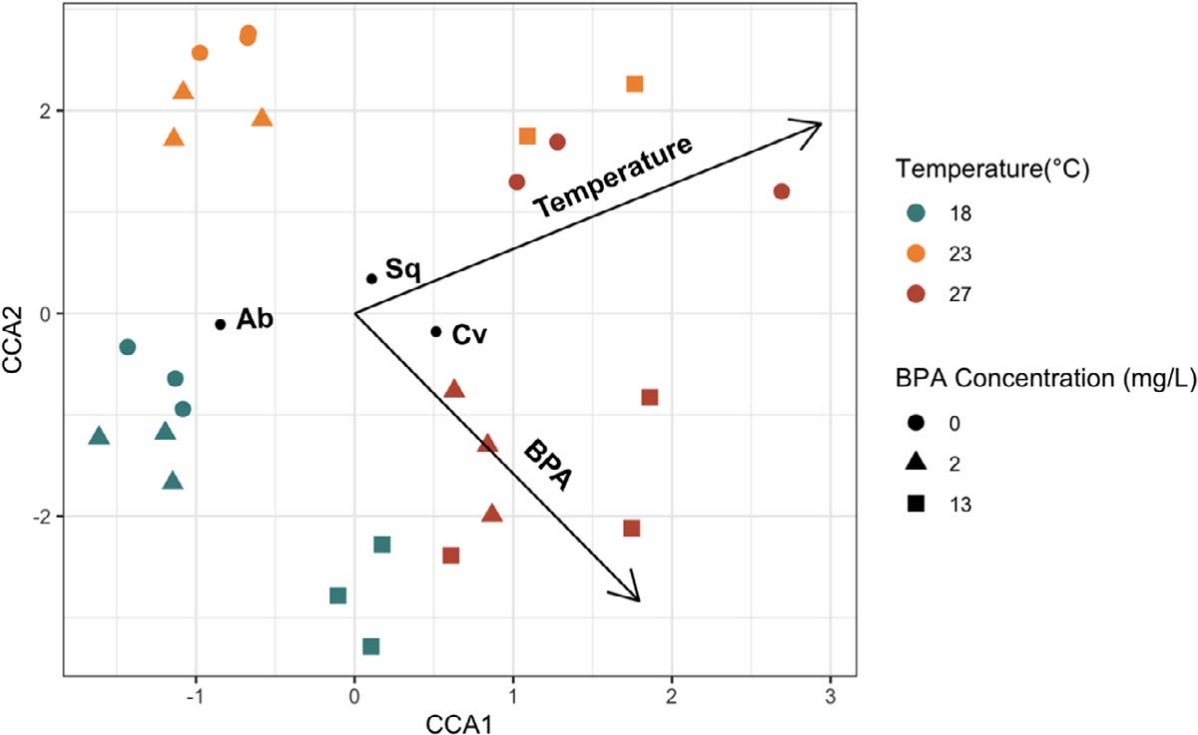
CCA plot (two dimensions) for multispecies assemblages exposed to various combinations of temperature (°C) and initial BPA concentration (mg/L). Species identities (Ab = *A. braunii*, Sq = *S. quadricauda*, Cv = *C. vulgaris*) are plotted near assemblages where the particular species was most prevalent. Vectors representing temperature (top vector) and BPA concentration (BPA; bottom vector) are included. Certain replicates were nearly identical, and thus the points representing those assemblages overlap.

Multispecies assemblages cultured under similar experimental conditions were more compositionally similar to one another than to the other experimental assemblages, with *C. vulgaris* dominating the composition of all assemblages (Figure 4). Both temperature and BPA significantly affected phytoplankton community structure (Figure 4; CCA permutation test temperature, F_1,23_ = 20.766, *P* = 0.001; BPA concentration, F_1,23_ = 11.063, *P* = 0.001; Supplementary Table S5). Assemblages cultured in 0 and in 2 mg/L BPA under 23°C were compositionally similar and were associated with the highest proportions of *S. quadricauda*. Assemblages cultured in 0 and in 2 mg/L BPA under 18°C were compositionally similar and were associated with the highest proportions of *A. braunii.* Under 27°C, the assemblages cultured in 2 mg/L BPA were more compositionally similar to the assemblages cultured in 13 mg/L BPA than those cultured in 0 mg/L BPA. The highest proportions of *C. vulgaris* were associated with assemblages cultured in 2 or 13 mg/L BPA under 27°C and in 13 mg/L BPA under 18°C. The greatest variation across replicates occurred in assemblages cultured under 27°C and in either 13 mg/L BPA or in 0 mg/L BPA.

## 4. Discussion

### 4.1. Consequences of BPA for phytoplankton growth rates

In contrast to our original hypothesis that BPA and stressfully warm temperatures would synergistically decrease growth rates, our results demonstrate that temperature and BPA may antagonistically interact to modify the response of particular phytoplankton species to stressfully warm temperatures (*A. braunii*) or to warmer temperatures that would otherwise lead to increase growth rates (*S. quadricauda*, *C. vulgaris*).

Although population growth in a majority of treatments was inhibited under 13 mg/L BPA, *A. braunii* and *S. quadricauda* populations were not negatively impacted by 2 mg/L BPA relative to 0 mg/L BPA. Thus, these results suggest that modest concentrations of BPA may not be detrimental in the short term for some phytoplankton species. BPA may provide a source of organic carbon (Wang *et al.,* 2017a) or may function to increase net photosynthetic rate and chlorophyll content, as seen in soybean seedlings exposed to similarly low levels of BPA (1.5 mg/L BPA) (Qiu *et al.,* 2013). However, if the rate of BPA uptake is far greater than the rate of BPA transformation, if cellular carbon requirements are already met or if the species is unable to use BPA, then BPA may rapidly accumulate in the cell, resulting in oxidative stress, lipid peroxidation and changes in metabolic enzyme activity (Ji *et al.,* 2014; Xiang *et al.,* 2018; Wu and Seebacher, 2021). These negative effects of BPA likely resulted in the reduced growth rates observed in the phytoplankton populations exposed to 13 mg/L BPA. Indeed, medium to high experimental BPA concentrations (7–50 mg/L BPA) have been shown to reduce the net photosynthetic rate and chlorophyll content in soybean seedlings, potentially due to the peroxidation of the membrane lipids of chloroplasts (Qiu *et al.,* 2013). Similarly, BPA concentrations >3 mg/L have been shown to reduce photosynthetic activity in the green algae species *Desmodesmus* (Wang *et al.,* 2017b). Differences in response to BPA between species may be due to differences in the metabolic processes involved in BPA uptake and degradation between phytoplankton species (Nakajima *et al.,* 2007; Wang *et al.,* 2017b). We encourage future research to investigate the effects of environmentally relevant BPA concentrations, which are typically <10 μg/L (Arnold *et al.,* 2013; Corrales *et al.,* 2015).

In *C. vulgaris* and *S. quadricauda* populations, growth rate generally increased as temperature increased. Contrastingly, in *A. braunii*, growth rate generally decreased as temperature increased in the absence of BPA. However, BPA antagonistically interacted with temperature in *A. braunii* because the negative effects of warmer temperatures were reduced in the presence of BPA. Although future research should address the effects of BPA on the thermal sensitivity of phytoplankton growth, our growth rate findings are consistent with previous research that indicates temperature can influence the rate of BPA uptake and excretion, the degree of bioaccumulation (Honkanen and Kukkonen, 2006; Wu and Seebacher, 2021) and the severity of the effects of BPA (Little and Seebacher, 2015). As temperature increased, *C. vulgaris* growth in 0 mg/L BPA became increasingly faster than growth in the presence of BPA, and in *S. quadricauda*, growth of populations cultured in 2 mg/L BPA and in 0 mg/L BPA were increasingly faster than growth of populations cultured in 13 mg/L BPA as temperature increased. In these phytoplankton species, stressful concentrations of BPA antagonistically interacted with temperature to reduce the positive effects of temperature. The rates of biochemical reactions typically increase exponentially with increasing temperature below the thermal optimum for the reaction pathway but decline above the optimum (Ritchie, 2018). Thus, as temperature changes, the efficiency of BPA uptake, transport (Honkanen and Kukkonen, 2006; Wu and Seebacher, 2020) and transformation may asynchronously respond due to differences in the thermal optimum and sensitivity of the associated reaction pathways and/or enzymes. Ultimately, the mechanisms behind the thermal specificity of BPA toxicity remain unknown, and if BPA disrupts multiple receptors or reaction pathways, then the differences in the sensitivity of these pathways to BPA may change with environmental conditions (Little and Seebacher, 2015). Nevertheless, the results presented in this study do suggest that the thermal specificity of BPA may not be solely due to its role as an endocrine disruptor because phytoplankton do not have endocrine systems.

### 4.2. Scaling from populations to multispecies phytoplankton assemblages

Although BPA, particularly at high concentrations, generally had negative consequences for the growth rate of phytoplankton, the community-level impacts of BPA were more nuanced. Our results confirm the ability of environmental temperature to modify the average phytoplankton size (Yvon-Durocher *et al.,* 2015); however, BPA did not have a large influence on the relationship between temperature and colony size. In the one species for which BPA influenced colony size (*A. braunii*), BPA and temperature antagonistically interacted to influence colony size, with BPA reducing the positive effects of temperature. In addition, the total phytoplankton biovolume at the end of the community assay was not systemically reduced in moderate or high BPA concentrations, indicating that BPA may target phytoplankton population growth rate yet have little to no effect on algal carrying capacity. This ability of biological communities to compensate for the effects of toxins despite negative population-level effects has been previously observed (Noël *et al.,* 2006) and further suggests that total standing biovolume may not predict the extent of freshwater BPA contamination. This may be particularly true if higher trophic levels are negatively impacted by BPA to a greater extent than phytoplankton (Huang *et al.,* 2015).

Warmer temperatures and BPA likely reduced phytoplankton assemblage evenness by favoring faster growing species, suggesting that a combination of both stressors could substantially alter dominance patterns in natural phytoplankton communities. Warmer temperatures can be associated with greater density-dependent effects (Brown *et al.,* 2004; Vasseur, 2020), and the experimental temperature that maximized growth for *S. quadricauda* and the fast-growing *C. vulgaris* was 27°C likely causing these assemblages to reach carrying capacity faster than the assemblages cultured under 18°C. Therefore, the proportion of slower growing species was reduced in the assemblages cultured under 27°C thus reducing evenness, whereas under the 23°C, the carrying capacity of the assemblages may have been higher, allowing for more growth of the *S. quadricauda* and *A. braunii* populations before resource limitations were realized. Relatedly, 23°C was not the experimental temperature that maximized growth for most species—except for the slow-growing species, *A. braunii*, in 0 mg/L BPA—which may have facilitated greater species evenness because most populations were experiencing some temperature-mediated limitations to growth but were still capable of achieving positive growth rates (Hammarlund and Harcombe, 2019).

Similarly, the differences in population growth rates due to BPA may explain the observed Pielou evenness index differences across BPA treatments. The 13 mg/L BPA was likely physiologically stressful for all species, but the high ‘baseline’ growth rate of *C. vulgaris* may have buffered against this stress, and any removal of BPA by *A. braunii* and *S. quadricauda* may have further facilitated *C. vulgaris* growth. Indeed, *A. braunii* and *S. quadricauda* populations were typically unaffected by 2 mg/L BPA. Similarly, in microbial communities exposed to metal working fluids, facilitation occurs when only a few species in the community can survive and/or grow (Piccardi *et al.,* 2019). In 2 mg/L BPA, if *A. braunii* and *S. quadricauda* cells were capable of efficiently using BPA molecules, then there would be less BPA to come into contact with and negatively affect the *C. vulgaris* cells. However, under 27°C, multispecies assemblages cultured in 2 mg/L were more compositionally similar to assemblages cultured in 13 mg/L BPA than in 0 mg/L BPA. Thus, although our results indicate that BPA and temperature synergistically interacted to reduce phytoplankton assemblage evenness under 27°C, future research should investigate the extent to which this reduction in evenness can result in meaningful ecological consequences such as reductions in trophic transfer or a reduced ability to tolerate additional environmental stressors (Vinebrooke *et al.,* 2004).

### 4.3. Implications for freshwater ecosystems

Overall, the results of this study indicate that regional (climate warming) and local (environmental contamination) global change processes interact to affect freshwater phytoplankton populations and communities. There is considerable heterogeneity in the rates of current and anticipated temperature change across freshwater ecosystems (O’Reilly *et al.,* 2015); however, many aquatic systems are predicted to experience sustained periods of warming, with summer surface water temperatures increasing at rates as high as 1.3°C·decade^−1^ in certain bodies of water (Adrian *et al.,* 2009; O’Reilly *et al.,* 2015; Woolway and Merchant, 2019). Thus, many freshwater organisms across the globe will continue to experience environmental warming while simultaneously interacting with other abiotic factors, such as contaminants. As such, it is encouraging that two of the three phytoplankton species we assayed exhibited increased growth rates at higher temperatures. Yet, certain combinations of directional warming and BPA concentration in freshwater ecosystems do antagonistically inhibit phytoplankton growth and reduce phytoplankton evenness which, in turn, may reduce aquatic resource use efficiency and primary productivity (Righetti *et al.,* 2019; Otero *et al.,* 2020). As such, geographic regions where exceedingly high concentrations of BPA pollution cooccur with high rates of predicted warming, such as Africa, South America and South Asia (King and Harrington, 2018; Wu and Seebacher, 2020; Wu and Seebacher, 2021), may experience declines in phytoplankton productivity and evenness.

The results from this study indicate that climate warming and environmental BPA contamination can, at extremely high BPA concentrations, jointly affect phytoplankton population dynamics and community structure with substantial consequences for the dominance of certain phytoplankton taxa. Importantly, besides the highest BPA concentrations, we did not observe a synergistic impact of high temperatures and high BPA concentrations negatively impacting phytoplankton populations. In addition, warming may counteract BPA-induced reductions in growth for certain phytoplankton species that are adapted to life in high-temperature environments. However, important questions remain regarding the ecological function of phytoplankton communities that are modified by both temperature and BPA. Future research should seek to generalize whether phytoplankton species exhibit trade-offs between tolerating warm temperatures versus high BPA concentrations, or whether warm-adapted species are generally more BPA tolerant (i.e. stress-induced community tolerance) (Vinebrooke *et al.,* 2004). Resolving such patterns would help guide which algal assemblages may be more susceptible to these joint environmental stressors and thus be a focus for future conservation efforts (Vinebrooke *et al.,* 2004).

## Funding

This work was supported by the Robert and Patricia Lawlor Carlson Student Opportunity Fund grant to M.E.T., the Reed College Department of Biology and National Science Foundation [DEB 1856415] to S.B.F.

## Author Contributions

M.E.T. conceived of the study; M.E.T. and S.B.F. designed experiments; M.E.T. performed all experiments; M.E.T., J.M. and S.B.F. analyzed the results and M.E.T. wrote the first draft of the manuscript with all authors contributing substantially to revisions.

## Data Availability

All data supporting this paper are publicly available in a GitHub Repository: https://github.com/mertheus1999/Interactive-effects-of-temperature-and-Bisphenol-A-on-phytoplankton-growth-and-community-structure

(Theus *et al.,* 2023).

## Conflict of Interest statement

The authors report no conflict of interest.

## Supplementary Material

Web_Material_coad021
