# Supplementary material for: Interactive effects of temperature and bisphenol A on phytoplankton growth and community structure
Source: Conserv Physiol. 2023 May 3;11(1):coad021. doi: 10.1093/conphys/coad021 (PMC10157778; doi:10.1093/conphys/coad021)
Supplement: Web_Material_coad021 [file web_material_coad021.zip › SI.pdf]

Supporting Information for the Online Edition

Interactive effects of temperature and Bisphenol A on phytoplankton growth and community structure

Index

Table S1: Biovolume equations associated with each experimental phytoplankton species.

Table S2: Three-way ANOVA results for the population assay.

Table S3: Three-way ANOVA results for the multispecies assemblage biovolume component of the community assay.

Table S4: Two-way ANOVA results for the Pielou's evenness component of the community assay.

Table S5: CCA results for the community assay.

Table S1. The geometric form and biovolume equation associated with each experimental phytoplankton species.

Species	Geometric Form	Biovolume Equation [A]	VisualSpreadsheet Biolume Measurement [B]
C. vulgaris	Sphere	$V = (4/3)*\pi*r^3$	Sphere
S. quadricauda	Prolate Spheroid (cells)	$V = \pi*r^2*h$	Cylinder
A. braunii	Sickle-Shaped Cylinder	$V \cong (\pi/6)*w^2*h$	Prolate Spheroid

A: The biovolume equation associated with each species of phytoplankton. The symbols used in the equations are as follows: V = biovolume, r = radius, w = width, and h = height of the cell. The equation used for *S. quadricauda* volume calculation was determined by manually calculating the volume of 10 *S. quadricauda* colonies by using the length (µm) and width (µm) measurements and using $V = (\pi/6)*w^2*h$ to determine the volume of the individual cells (Sun and Liu 2003). The volumes of the individual cells were then totaled, and the total calculated volume was compared to the volumes calculated by the VisualSpreadsheet program (sphere, prolate spheroid, or cylinder). The cylinder volume determination of the VisualSpreadsheet program was most similar to the calculated *S. quadricauda* colony volume for 9 of the 10 tested colonies (average difference of 144.35 μm^3; standard deviation of the difference of 3,250.92 μm^3), thus, the cylinder volume was used to determine all *S. quadricauda* volumes. The biovolume equations for the *C. vulgaris* and *A. braunii* cells and colonies were not tested due to the consistency in the respective geometric form of each species across cells and colonies.

B: The VisualSpreadsheet program connected to the FlowCam 5000 automatically calculates the volume of each imaged particle using 3 biovolume equations associated with the following

geometric forms: sphere, prolate spheroid, and cylinder. The program assumes the imaged particle is the selected geometric form and calculates volume using the associated equation and length and width measurements. Thus, these equations may overestimate or underestimate the actual biovolume of the cells. The biovolume equation used for each species was manually chosen based on the most prevalent geometric form of the species in order to minimize overestimation and underestimation of biovolume.

Table S2. Three-way ANOVA to determine if temperature, BPA concentration, species identity, or the interaction of these factors affected phytoplankton growth rate. Results were determined to be statistically significant based on $\alpha = 0.05$.

**Predictor**	***df***	**Sum Square**	**Mean Square**	***F***	***P***
BPA Concentration	2	1.62	0.810	14.079	1.20×10^{-5}
Temperature	2	1.15	0.574	9.976	0.000206
Species	2	40.73	20.367	353.859	$< 2 \times 10^{-16}$
Species x BPA Concentration	4	1.50	0.374	6.498	0.000241
Species x Temperature	4	3.69	0.923	16.035	1.03×10^{-8}
Temperature x BPA Concentration	4	0.32	0.081	1.410	0.243137
Species x Temperature x BPA Concentration	8	1.88	0.236	4.094	0.000723
Residuals	54	3.11	0.058		

Table S3. Three-way ANOVA to determine if temperature, BPA concentration, species identity, or the interaction of these factors affected colony biovolume (μm^3).

Predictor	*df*	Sum Square	Mean Square	*F*	*P*
BPA Concentration	2	1128923	564461	0.628	0.5377
Temperature	2	98677934	49338967	54.903	<0.001
Species	2	446518484	223259242	248.437	<0.001
Species x BPA Concentration	4	7743493	1935873	2.154	0.0875
Species x Temperature	4	83900517	20975129	23.341	<0.001
Temperature x BPA Concentration	4	4197403	1049351	1.168	0.3360
Species x Temperature x BPA Concentration	8	12400265	1550033	1.725	0.1151
Residuals	51	45831441	898656		

Table S4. Two-way ANOVA to determine if temperature, BPA concentration, or the interaction of temperature and starting BPA concentration affected the log Pielou's evenness index in the community assay. Results were determined to be statistically significant based on $\alpha = 0.05$.

**Predictor**	***df***	**Sum Square**	**Mean Square**	***F***	***P***
BPA Concentration	2	11.49	5.74	52.717	<0.001
Temperature	2	17.29	79.31	79.31	<0.001
Temperature x BPA	4	1.77	4.06	4.058	0.017
Residuals	17	1.852	0.11		

Table S5. CCA results for the community assay. Results were determined to be statistically significant based on $\alpha = 0.05$.

**Predictor**	***df***	X^2	***F***	***P***
Temperature	1	0.00240	20.766	0.001
BPA Concentration	1	0.00128	11.063	0.004
Residuals	23	0.00266		
